# Sex Differences in Pain Sensitivity in a Dutch Cohort: Cross-Sectional and Web-Based Multidimensional Study

**DOI:** 10.2196/53926

**Published:** 2024-08-09

**Authors:** Rachel J H Smits, Selina E I van der Wal, Regina L M van Boekel, Hans Timmerman, Ewald M Bronkhorst, Diana Abrar, Kris C P Vissers, Esmeralda N Blaney Davidson, Monique A H Steegers

**Affiliations:** 1 Department of Anesthesiology, Pain and Palliative Medicine Radboud university medical center Nijmegen Netherlands; 2 Department of Anesthesiology, Pain Center University of Groningen University Medical Center Groningen Groningen Netherlands; 3 Department for Health Evidence Radboud university medical center Nijmegen Netherlands; 4 Department of Rheumatic Diseases Radboud university medical center Nijmegen Netherlands; 5 Department of Anesthesiology Amsterdam University Medical Center, location VU Medical Center Amsterdam Netherlands

**Keywords:** pain sensitivity, pain sensitivity questionnaire, chronic pain, digital interventions, mobile health, film, sex, Dutch population, personalized medicine, individualized care

## Abstract

**Background:**

Sex is an important factor influencing the development and treatment of chronic pain, but the extent of its influence is still unclear. Other demographic factors as well as nonpharmacological interventions might influence pain sensitivity differently in men and women.

**Objective:**

In this study, we aimed to investigate the influence of sex and other demographic, lifestyle, behavioral, clinical, and environmental factors on pain sensitivity in the Dutch population. Different films were used to investigate how they would impact pain sensitivity and what influence sex and other variables have on the effect of this simple intervention.

**Methods:**

We performed a study consisting of 2 parts: (1) a cross-sectional research to investigate pain sensitivity differences between men and women and the influence of other demographic variables on the pain sensitivity in a Dutch cohort and (2) an internet intervention study to determine whether a short film could skew pain sensitivity.

**Results:**

All respondents filled in a web-based demographic questionnaire and were randomized into 4 groups. The control group filled in the Pain Sensitivity Questionnaire without watching a preliminary film. A cross-sectional analysis was performed in the control group (n=1746). The other 3 groups watched short films: one group watched a film with scenes of nature (n=2650), another group watched a film on laughing people (n=2735), and the last group watched a film on physically painful events (n=2708). Immediately after the film viewing, participants were directed to the Pain Sensitivity Questionnaire to measure their pain sensitivity. The Pain Sensitivity Questionnaire score was stated as a mean per question on the numeric rating scale from 0-1. The cross-sectional study revealed no significant differences between men and women but showed male-female differences in the Pain Sensitivity Questionnaire when specific background factors were present. Watching a short film had a positive impact on the pain sensitivity of the respondents who had chronic pain, with a higher effect observed in female respondents.

**Conclusions:**

Scientists performing pain research need to account for factors that can influence the outcome of their study and be aware that these factors can be sex-dependent, and pain sensitivity should be analyzed accordingly. Even relatively small interventions such as watching a film can impact pain sensitivity, especially in respondents with current chronic pain. This effect can vary as well when different background factors are present. Our findings warrant further explorations of the possibilities that simple interventions bring for patients in personalized medicine.

**Trial Registration:**

Landelijk Trial Register NTR-new NL8182; https://onderzoekmetmensen.nl/en/trial/29537

## Introduction

According to the International Association for the Study of Pain, pain is a personal experience that is influenced in varying degrees by biological, psychological, and social factors [1]. Individual pain sensitivity is an important factor in the experience and treatment of pain. A higher pain sensitivity is associated with the development of chronic pain [2]. Numerous demographic variables such as age, obesity, and smoking have been associated with the development of chronic pain [3-5]. Analgesic drug treatment of chronic pain is often difficult, insufficient, and has substantial side effects, thereby emphasizing the need for more nonpharmacological preventive and treatment options [6,7].

Sex is an important factor influencing chronic pain as well [8,9]. Women report a higher prevalence of chronic pain and experience more severe and longer lasting pain compared with men in comparable situations [10,11]. Important variability has been observed in the pharmacokinetics and pharmacodynamics between sexes, with a higher analgesic effect and more side effects of opioids and other pharmacological agents in women [12,13]. Men and women have also different responses to treatment in multimodal pain management and cognitive behavioral therapy [14,15]. However, the effects of sex and other background factors on pain sensitivity and whether women or men are more sensitive to painful stimuli are unclear [16]. Moreover, demographic, lifestyle, behavioral, clinical, and environmental factors might influence pain sensitivity differently in men and women [9]. These sex differences can be linked to specific physiological mechanisms such as cerebral responses to visual, auditory, emotional, and pain processing stimuli [17]. This includes the observation of the strong influence of empathy in experimental settings on pain and higher thresholds for painful stimuli after social laughter among women [18,19]. These factors raise the question whether nonpharmacological interventions such as watching a short film might work differently between men and women.

With this study, we wanted to raise awareness in the Dutch population regarding chronic pain and the sex differences in chronic pain. With our data, we aimed to investigate the influence of sex and other demographic, lifestyle, behavioral, clinical, and environmental factors on pain sensitivity. Short films with different contents were implemented to make the study more appealing for the public and were used to investigate how films would impact pain sensitivity and what influence sex and other variables have on the effect of this simple intervention.

## Methods

### Recruitment

In 2017 and 2018, a national campaign was launched in the Netherlands in cooperation with the Dutch Public Broadcast Corporation (Nederlandse Publieke Omroep) to raise awareness of chronic pain and sex differences in chronic pain. This study was part of this campaign and enabled people to actively participate and gain knowledge about pain and their own pain sensitivity. This awareness creation included a broadcast on national television, several radio performances, and promotion at festivals. The website was presented through the national campaign. Volunteers were enrolled between May 4, 2017, and October 8, 2018.

### Ethics Approval

This study adheres to the applicable CONSORT (Consolidated Standards of Reporting Trials) guidelines. The trial protocol was approved by the Medical Ethical Committee on Research Involving Human Subjects (Arnhem-Nijmegen NL2017-3314). The study was registered in the Dutch national trial register on November 21, 2019 (NTR-new NL8182).

### Trial Oversight

This first Dutch national web-based pain research trial in the general population was a large national mixed methods study with a cross-sectional questionnaire in the Dutch population and a concomitant feasibility study with a web-based intervention. The trial was designed by a committee formed at the Radboud University Medical Center. It was supported and partly funded by the Dutch Society for Scientific Research and an independent Dutch public service broadcaster specializing in information, education, and culture. The funders of the study had no role in study design, data collection, data analysis, data interpretation, or writing of the report.

This study was performed in collaboration with Dutch television. This enabled us to gather data on a very large population but was associated with a tight deadline in which we had to move very quickly to get the study up and running. Therefore, our entire team was focused on ensuring that the setup of our experiment was sound, but as a result, the registration was delayed. The protocol was not altered after the gained approval by the Medical Ethical Committee, and the website was launched on May 4, 2017. No adjustments were made in the website until the last inclusion.

### Population

Adults with proper understanding of the Dutch language could voluntarily participate in this study. The informed consent procedure was performed digitally. A total of 10,876 consenting volunteers participated. Filling in the demographic questionnaire was optional. After excluding the participants who did not complete the demographic questionnaire and those who gave impossible answers, 9839 respondent data were analyzed.

### Trial Procedure

After registration, participants were requested to fill out a general baseline questionnaire containing demographic factors and variables that might influence pain sensitivity. These included sex, age, weight, height, highest level of education, smoking, hours of physical activity per week, current pain level on a numeric rating scale (NRS 0: no pain to 10: worst pain imaginable), and the duration of the current pain. They were not obligated to fill in information and were allowed to skip questions; only participants who completed the questionnaires about demographics and pain sensitivity were included in the final database for the cross-sectional analysis.

**Figure 1:**
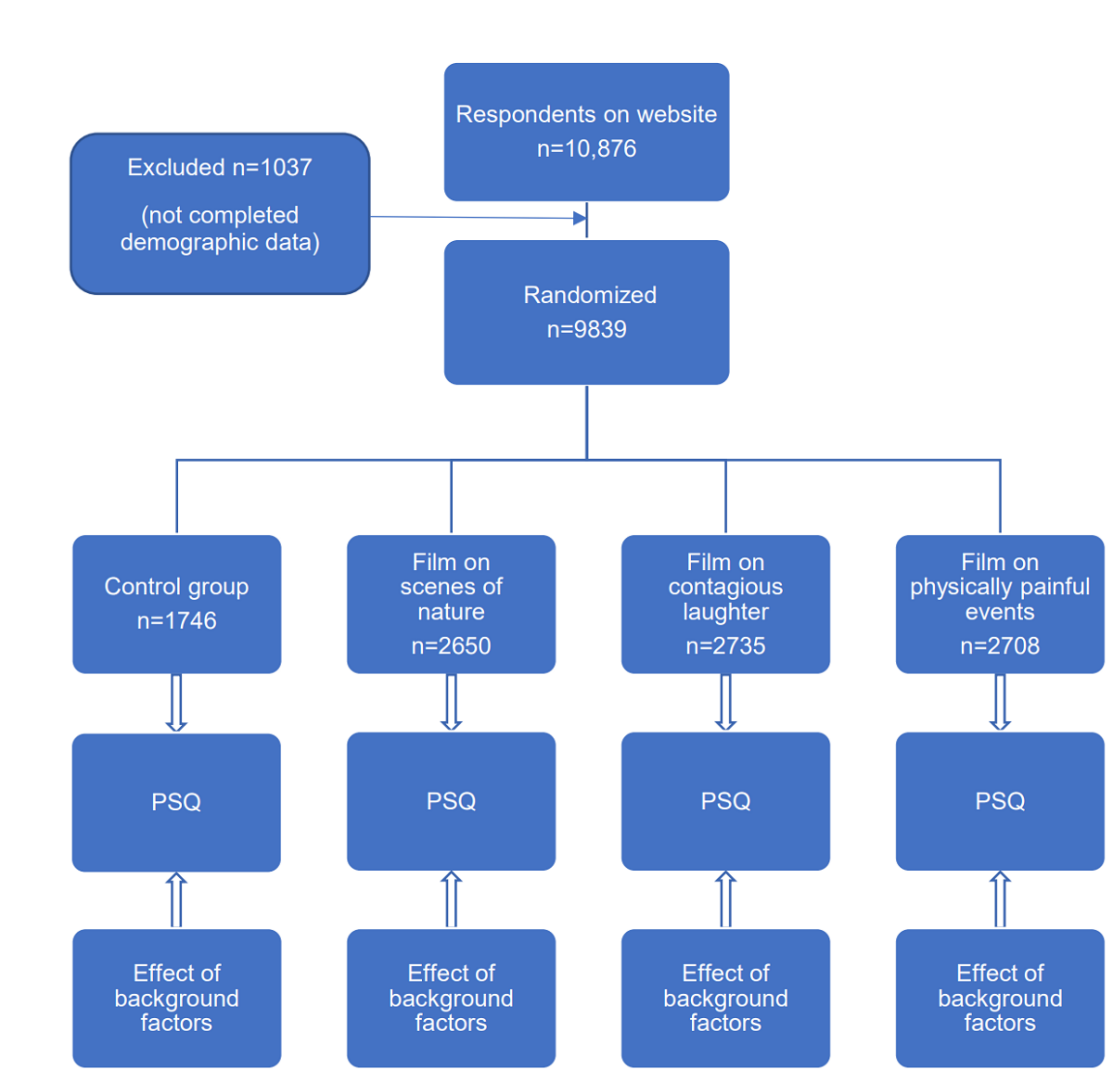
Enrollment and randomization of respondents. PSQ: Pain Sensitivity Questionnaire.

After completion of the baseline questionnaire, a built-in automated complete blind randomization program allocated the respondents into 1 of the 4 groups (Figure 1). The control group filled in the Pain Sensitivity Questionnaire (PSQ) without watching a preliminary film. These data were used for the cross-sectional analysis. The other groups were shown one of the 3 films with a duration of 45 seconds. The clips in the short films were chosen via a discussion-based focus group. The first film was considered as a neutral film, displaying calm scenes of nature like gently flowing water; the second film showed contagious laughter of people of all ages; and the third film showed people of all ages enduring clearly physically painful situations. Immediately after the viewing, participants were directed to the PSQ to measure their pain sensitivity. The feasibility study was designed to assess whether a simple intervention such as watching a film could influence pain sensitivity, as measured using PSQ.

### Study Outcome

The primary outcome of this study was the score on PSQ, reflecting the participant’s pain sensitivity and the effect of background factors on their pain sensitivity. The PSQ was developed by Ruscheweyh and coworkers [20] to measure pain sensitivity in daily clinical practice without experimental determination of one’s pain. The validity to measure perceived pain sensitivity has been shown by significant correlations with experimental pain intensity in healthy individuals as well as in patients with chronic pain [20-22]. It has been used, among other instruments, to identify pain sensitivity in epidemiological health research and in several studies regarding postoperative and chronic pain before and after multiple surgeries [20-23].

The PSQ has been translated and validated in the healthy Dutch population [24]. It is a self-rating pain measurement tool consisting of 17 questions, which entail imagining potentially painful everyday life situations, and takes 5-10 minutes to complete. Respondents score their pain intensity of the described situation ranging from 0 (not painful) to 10 (strongest pain imaginable) on the NRS. Fourteen questions represent life situations that a majority of healthy individuals consider painful. Three questions represent situations that are generally perceived as nonpainful and are used as a nonpainful sensory reference not included in the final scoring. A higher score on the PSQ (range 0-140) corresponds to a higher sensitivity to pain, meaning the respondent rates a higher pain score for the same situation compared to a respondent with a lower score. To facilitate the interpretation of PSQ, the overall score is transformed to a score between 0 and 10; therefore, its score has the same range as the individual questions.

### Statistical Analysis

In the descriptive analyses, all categorical variables were presented with counts and percentages, while the continuous variables were presented with mean and range. The effect, stated as β, was presented with a 95% CI. To analyze the relation between sex and pain sensitivity, adjusted for other background factors, a multiple linear regression model was used. In this model, full interaction between sex and all other demographic variables was added, without demand for statistical significance. Because of the large sample size, power analysis was not performed. For the same reason, we used the model as specified, without checking for multicollinearity, relying on the large sample size to compensate for potential loss of precision due to multicollinearity. The model was fully created with an “enter philosophy” in mind; all variables were entered at once.

For the feasibility study concerning the effect of exposure to various short films on the PSQ score, linear regression was applied again. Due to the smaller sample size in the control group compared to the other group sizes, the film on the calm scenes of nature was used as the control group (as a neutral audiovisual stimulus) to compare the effects of the film on contagious laughter and the film on painful events to limit the chance of subtle selection effects. Film was entered in the model as a categorical variable, with nature film as a reference. In addition to film, sex was added to the model as well, supplemented with an interaction term for sex and film.

Based on the relations found in the demographic analysis, a more comprehensive analysis of the effect of the film was undertaken. In this analysis, the base regression model was a 3-way interaction between film, sex, and NRS score, supplemented with all other demographic variables. Interactions with this last group of variables were only added if it improved the model fit significantly. Analysis was performed using SPSS Statistics for Windows (version 25.0; IBM Corp; released 2013) and R (version 3.6.2). In all analyses a *P* value <.05 was considered statistically significant.

## Results

### Cross-Sectional Analysis Results

The common variables gathered in demographic research included age, sex, BMI, smoking, exercise in hours per week, and education (Table 1). Of the 7375 female respondents, 25.9% (1908/7375) reported no pain, 27.3% (2016/7375) reported mild pain, 35.2% (2599/7375) reported moderate pain, and 10.9% (810/7375) reported severe pain. In women experiencing pain, 81.2% (4403/5425) had pain lasting more than 3 months (eg, chronic pain). In the male cohort, 39.8% (979/2464) did not report pain, 31.5% (775/2464) reported mild pain, 23.2% (571/2464) reported moderate pain, and 5.5% (134/2464) reported severe pain. The pain was present for more than 3 months in 74.8% (1111/1485) of the men reporting pain.

**Table 1:** Complete demographic data of all the respondents (N= 9839).

		No intervention					Nature film					Contagious laughter film					Painful event film		
		Males (n=411)		Females (n=1335)		Males (n=625)			Females (n=2025)		Males (n=700)			Females (n=2035)		Males (n=728)			Females (n=1980)
Age (years), mean (range)		49 (18-89)		44 (18-93)		49 (18-91)			44 (18-86)		49 (18-90)			44 (18-88)		50 (18-90)			43 (18-85)
BMI (kg/m²), mean (range)		25.0 (16.5-46.8)		25.9 (14.3-52.4)		25.2 (17.1-43.3)			25.5 (14.2-62.4)		25.5 (15.6-56.8)			25.6 (13.9-61.3)		25.5 (15.7-56.4)			25.8 (14.0-61.0)
**Smoking, n (%)**																			
	Yes	60 (14.6)	226 (16.9)		95 (15.2)			336 (16.6)		116 (16.6)			313 (15.4)		131 (18)			312 (15.8)	
	No	350 (85.2)	1104 (82.7)		529 (84.6)			1683 (83.1)		583 (83.3)			1716 (84.3)		592 (81.3)			1658 (83.7)	
	Missing	1 (0.2)	5 (0.4)		1 (0.2)			6 (0.3)		1 (0.1)			6 (0.3)		5 (0.7)			10 (0.5)	
**Practice sports, n (%)**																			
	Yes	224 (54.5)	721 (54)		353 (56.5)			1098 (54.2)		397 (56.7)			1156 (56.8)		399 (54.8)			1085 (54.8)	
	No	185 (45)	609 (45.6)		270 (43.2)			920 (45.4)		303 (43.3)			873 (42.9)		329 (45.2)			892 (45)	
	Missing	2 (0.5)	5 (0.4)		2 (0.3)			7 (0.4)		0 (0)			6 (0.3)		0 (0)			3 (0.2)	
**Exercise (h/wk), n (%)**																			
	No sport	185 (45)	609 (45.6)		270 (43.2)			920 (45.4)		303 (43.3)			873 (42.9)		329 (45.2)			892 (45)	
	0-1	29 (7.1)	128 (9.6)		48 (7.7)			230 (11.3)		47 (6.7)			220 (10.8)		45 (6.2)			212 (10.7)	
	2-3	88 (21.4)	386 (29)		149 (23.8)			541 (26.7)		172 (24.6)			593 (29.1)		181 (24.9)			582 (29.4)	
	3-6	77 (18.7)	157 (11.7)		105 (16.8)			259 (12.8)		112 (16)			261 (12.8)		111 (15.2)			211 (10.7)	
	7-10	20 (4.9)	37 (2.8)		41 (6.6)			50 (2.5)		49 (7)			64 (3.1)		48 (6.6)			64 (3.2)	
	More than 10	10 (2.4)	12 (0.9)		11 (1.7)			16 (0.8)		17 (2.4)			17 (0.8)		14 (1.9)			14 (0.7)	
	Missing	2 (0.5)	6 (0.4)		1 (0.2)			9 (0.5)		0 (0)			7 (0.3)		0 (0)			5 (0.3)	
**Education, n (%)**																			
	Primary school	7 (1.7)	26 (1.9)		13 (2.1)			38 (1.9)		14 (2)			46 (2.3)		16 (2.2)			44 (2.2)	
	Secondary school	63 (15.3)	244 (18.3)		97 (15.5)			374 (18.5)		123 (17.6)			356 (17.5)		125 (17.2)			370 (18.7)	
	Secondary vocational school	84 (20.4)	381 (28.5)		134 (21.4)			556 (27.5)		158 (22.6)			551 (27.1)		161 (22.1)			540 (27.3)	
	Higher professional education or up	249 (60.6)	649 (48.6)		364 (58.2)			1015 (50.1)		395 (56.4)			1048 (51.5)		415 (57)			977 (49.3)	
	No education	3 (0.7)	19 (1.4)		11 (1.8)			25 (1.2)		4 (0.6)			24 (1.2)		11 (1.5)			27 (1.4)	
	Missing	5 (1.2)	16 (1.2)		6 (1)			17 (0.8)		6 (0.8)			10 (0.5)		0 (0)			22 (1.1)	
**Current pain score (numeric rating scale score), n (%)**																			
	No pain	183 (44.5)	314 (23.5)		240 (38.4)			538 (26.6)		293 (41.9)			533 (26.2)		263 (36.1)			523 (26.4)	
	Mild (1-3)	120 (29.2)	337 (25.2)		211 (33.8)			536 (26.5)		198 (28.3)			583 (28.6)		246 (33.8)			560 (28.3)	
	Moderate (4-7)	84 (20.5)	500 (37.5)		138 (22.1)			723 (35.7)		175 (25)			704 (34.6)		174 (23.9)			672 (33.9)	
	Severe (8-10)	24 (5.8)	177 (13.3)		34 (5.4)			215 (10.6)		33 (4.7)			204 (10)		43 (5.9)			214 (10.8)	
	Missing	0 (0)	7 (0.5)		2 (0.3)			13 (0.6)		1 (0.1)			11 (0.5)		2 (0.3)			11 (0.6)	
**Duration of pain (months), n (%)**																			
	No pain	183 (44.5)	314 (23.5)		240 (38.4)			538 (26.6)		293 (41.9)			533 (26.2)		263 (36.1)			523 (26.4)	
	Shorter than 3 months	55 (13.4)	155 (11.6)		95 (15.2)			278 (13.7)		91 (13)			297 (14.6)		113 (15.5)			258 (13)	
	3-6 months	17 (4.2)	62 (4.6)		25 (4)			71 (3.5)		26 (3.7)			84 (4.1)		36 (4.9)			65 (3.3)	
	Longer than 6 months	151 (36.7)	788 (59)		257 (41.1)			1120 (55.3)		286 (4.8)			1098 (53.9)		313 (43)			1115 (56.3)	
	Missing	5 (1.2)	16 (1.2)		8 (1.3)			18 (0.9)		4 (0.6)			23 (1.1)		3 (0.4)			19 (1)	

### Pain Sensitivity Is Influenced by Background Factors

**Figure 2:**
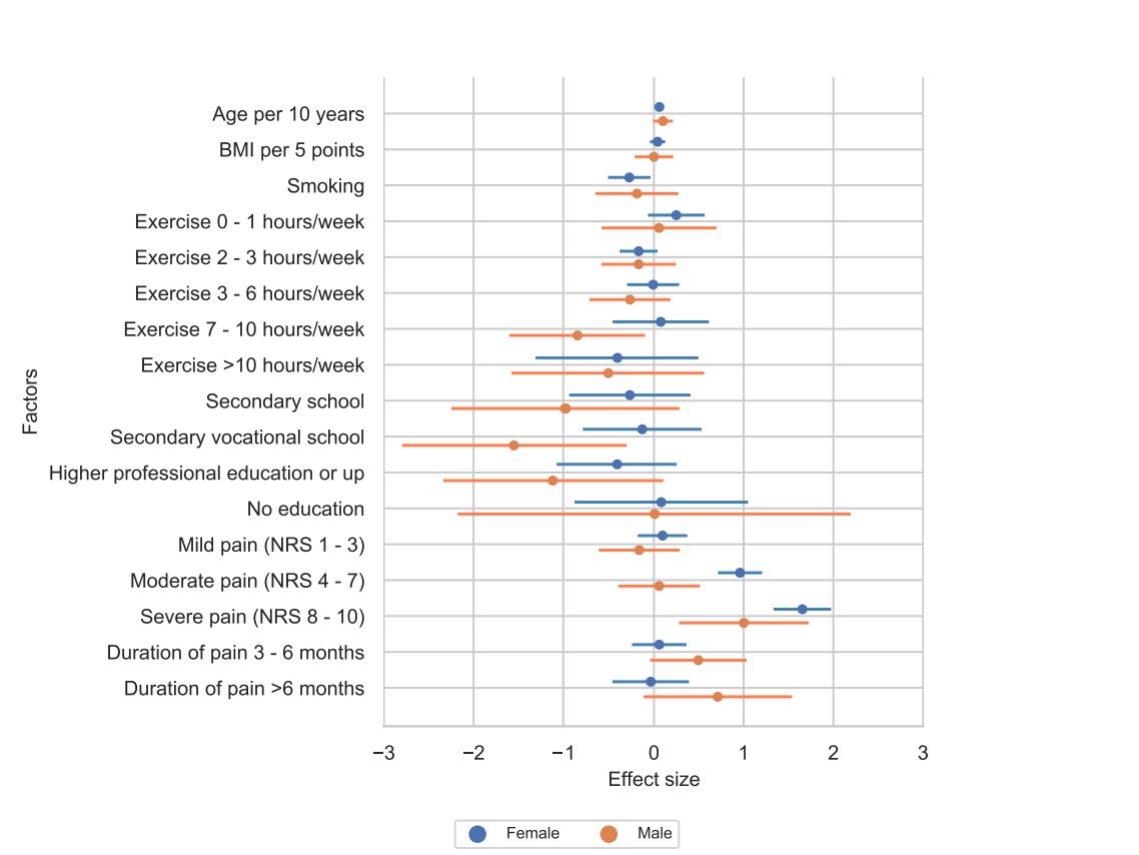
Effects of different background factors on the Pain Sensitivity Questionnaire scores of men and women. NRS: numeric rating scale.

A total of 1746 respondents filled in the PSQ in the control group. This group was also used for cross-sectional analysis. The control group consisted of 411 men and 1335 women. Initially, we intended to analyze men versus women in a relatively simple manner and hypothesized their PSQ scores would be different. Overall, it was not possible to state whether the pain sensitivity is different in men and women without taking the background factors into account. In doing so, we found that factors that affect the PSQ scores positively were different between men and women. The PSQ scores of women who smoked were lower than those who did not smoke (–.28). Men with a higher education had a lower PSQ score, while for women, no difference was seen in the PSQ score between different levels of education. Experiencing moderate and severe pain in women affected the PSQ scores negatively (.96 and 1.65, respectively), meaning a higher score on the PSQ. Men with severe pain also had a higher score on the PSQ, but this result was obtained in a very small group (n=24). Table 2 and Figure 2 show the effect of different background factors on the PSQ scores in men and women.

**Table 2:** Effect of various background factors on the Pain Sensitivity Questionnaire scores in men and women in the control group (N=1746).

Background factors per category		Males				Females		
		Value	β (95% CI)	*P* value	Value		β (95% CI)	*P* value
Total (intercept), n		411	4.38 (2.76 to 6.00)	<.001	1335		3.48 (2.64 to 4.33)	<.001
Sex, n		411	–.90 (–2.72 to .93)	.34	1335		.90 (–.93 to 2.72)	.34
Age (years), mean (range)		49 (18 to 89)	.01 (.00 to .02)	.06	44 (18 to 93)		.01 (.00 to .01)	.07
BMI (kg/m²), mean (range)		25.0 (16.5 to 46.8)	0 (–.04 to .04)	.99	25.9 (14.3 to 52.4)		.01 (–.01 to .03)	.32
**Smoking, n (%)**								
	No	350 (85.2)	Ref^a^	Ref	1104 (82.7)		Ref	Ref
	Yes	60 (14.6)	–.19 (–.65 to .27)	.42	226 (16.9)		–.28 (–.51 to –.04)	.02
	Missing	1 (0.2)	N/A^b^	N/A	5 (0.4)		N/A	N/A
**Exercise (h/wk), n (%)**								
	No sport	185 (45)	Ref	Ref	609 (45.6)		Ref	Ref
	0-1	29 (7.1)	.06 (–.58 to .70)	.86	128 (9.6)		.25 (–.07 to .57)	.13
	2-3	88 (21.4)	–.17 (–.59 to .24)	.42	386 (29)		–.17 (–.38 to .04)	.11
	3-6	77 (18.7)	–.27 (–.72 to .18)	.25	157 (11.7)		–.01 (–.30 to .28)	.95
	7-10	20 (4.9)	–.85 (–1.60 to –.10)	.03	37 (2.8)		.08 (–.46 to .61)	.78
	>10	10 (2.4)	–.51 (–1.58 to .56)	.35	12 (0.9)		–.41 (–1.31 to .50)	.38
	Missing	2 (0.5)	N/A	N/A	6 (0.4)		N/A	N/A
**Education, n (%)**								
	Primary school	7 (1.7)	Ref	Ref	26 (1.9)		Ref	Ref
	Secondary school	63 (15.3)	–.98 (–2.25 to .28)	.13	244 (18.3)		–.27 (–.95 to .41)	.44
	Secondary vocational school	84 (20.4)	–1.55 (–2.80 to –.31)	.02	381 (28.5)		–.13 (–.79 to .53)	.70
	Higher professional education or up	249 (60.6)	–1.12 (–2.34 to .10)	.07	649 (48.6)		–.41 (–1.08 to .25)	.23
	No education	3 (0.7)	.01 (–2.18 to 2.19)	.99	19 (1.4)		.08 (–.89 to 1.05)	.87
	Missing	5 (1.2)	N/A	N/A	16 (1.2)		N/A	N/A
**Current pain score (numeric rating scale score), n (%)**								
	No pain	183 (44.5)	Ref	Ref	314 (23.6)		Ref	Ref
	Mild (1-3)	120 (29.2)	–.16 (–.62 to .29)	.48	337 (25.4)		.10 (–.18 to .37)	.50
	Moderate (4-7)	84 (20.5)	.06 (–.40 to .51)	.80	500 (37.7)		.96 (.71 to 1.20)	<.001
	Severe (8-10)	24 (5.8)	1.00 (.28 to 1.72)	.01	177 (13.3)		1.65 (1.34 to 1.97)	<.001
	Missing	0 (0)	N/A	N/A	7 (0.1)		N/A	N/A
**Duration of pain (months), n (%)**								
	No pain	183 (44.5)	Ref	Ref	314 (23.5)		Ref	Ref
	Shorter than 3 months	55 (13.4)	.50 (–.04 to 1.03)	.07	155 (11.6)		.06 (–.24 to .36)	.70
	Longer than 3 months	168 (40.9)	.71 (–.12 to 1.54)	.09	850 (63.6)		–.04 (–.46 to .39)	.87
	Missing	5 (1.2)	N/A	N/A	16 (1.2)		N/A	N/A

^a^Ref: reference.

^b^N/A: not applicable.

To clarify the results, we used a model in the form of a hypothetical 40-year-old nonsmoking respondent with a BMI of 25, who had no pain, exercises 2-3 h/wk, and had completed higher education. If this respondent was a female, she had an estimated PSQ score of 3.34. If this respondent was a male, his estimated PSQ score was 3.51 in this model. However, if this respondent had the same demographic characteristics but was experiencing moderate pain at the moment, the PSQ score for a female respondent would be 4.29, while that for the male respondent would be 3.57. Further, if this respondent was experiencing severe pain, the PSQ score was higher in both sexes compared to the respondents without pain or with moderate pain (female 4.99, male 4.52). This exemplifies how various factors can influence pain sensitivity differentially in men and women.

### Film and Pain Sensitivity

**Figure 3:**
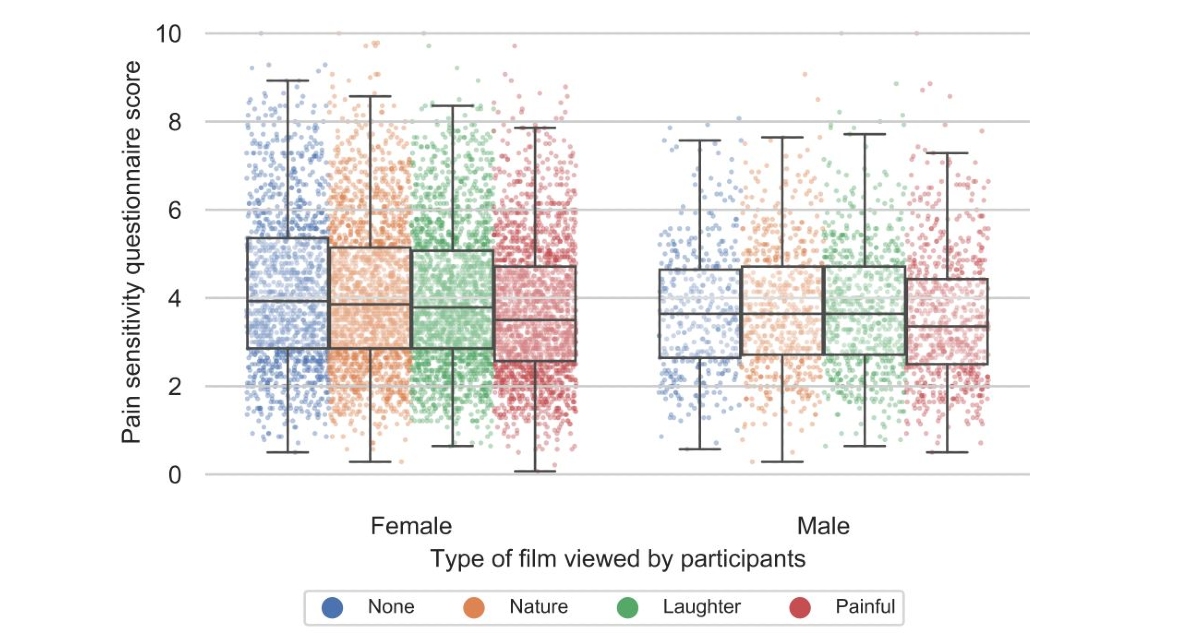
Effects of the types of films on the Pain Sensitivity Questionnaire scores of men and women.

The distribution of demographics in the control and nature film groups was similar due to randomization, and there was no difference in the PSQ scores between the group that saw a film with nature and those who did not see a film before filling in the PSQ, which makes it possible to use the nature film group as a neutral film and reference, thereby overcoming the limitation of the smaller sample size of the control group. When considering the complete group, the film with contagious laughter had no significant effect on the PSQ scores in men or women. In the group of respondents who watched the film in which people endured painful situations, both men and women documented a lower score on the PSQ (–.20 for men and –.37 for women) (Figure 3). Even though the difference between men and women was not statistically significant (*P*=.07), the outcome of the differential impact that background variables have on men and women made us decide to investigate whether this would translate into a different effect of the films as well. This revealed that even the effect of a short film on pain sensitivity is dependent on some background factors and that this differs between sexes.

**Figure 4:**
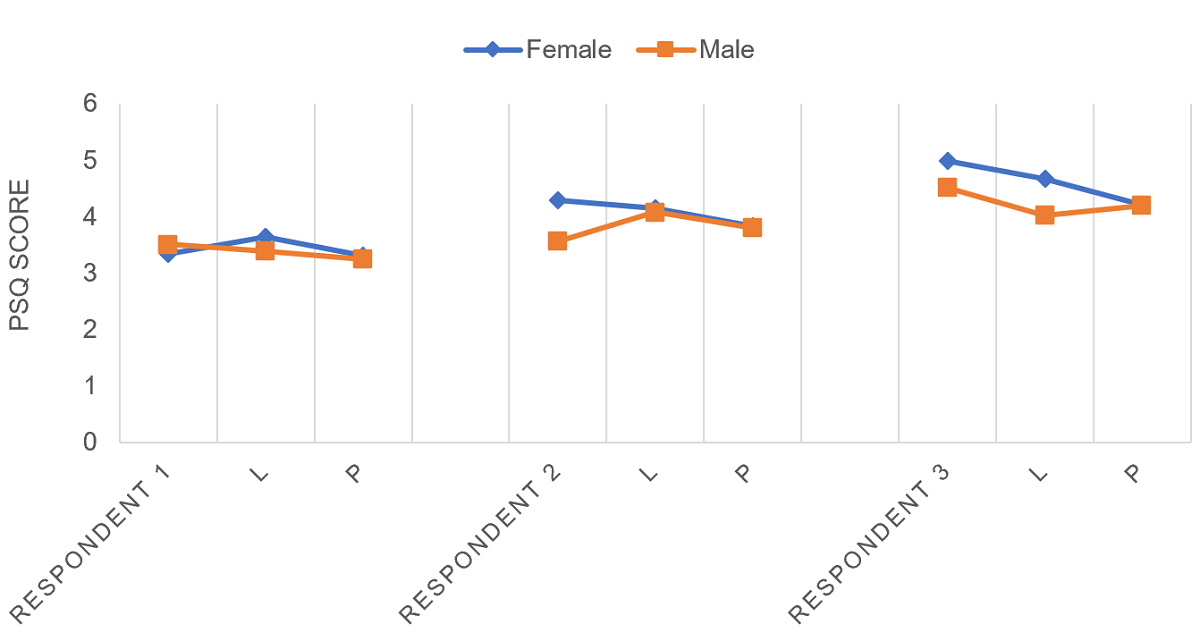
Scores of hypothetical respondents on the Pain Sensitivity Questionnaire. L: film on laughter; P: film on painful events; PSQ: Pain Sensitivity Questionnaire.

Sex, BMI, and (the degree of) current pain increased the effect of contagious laughter and painful event films. Sex and current pain were both influential factors. The combination of being female and experiencing pain had an additional lowering effect on the PSQ score. The other background factors that showed effect on the pain sensitivity did not influence the effect of the films on pain sensitivity. When circling back to the prior used hypothetical 40-year-old respondent (nonsmoking, with a BMI of 25, no current pain, exercise of 2-3 h/wk, and a higher education), we only see a minimal difference in the PSQ score between both female (3.31) and male (3.25) respondents after watching a painful event film (Figure 4, respondent 1). However, the film with painful situations had a larger positive effect in the model when the respondent was experiencing moderate pain at the moment (female 3.84, male 3.80, Figure 4, respondent 2). In the respondent who experienced severe pain, both sexes had a lower score on the PSQ after watching the contagious laughter film (female 4.68, male 4.02) and after watching the painful event film (female 4.21, male –4.20, Figure 4, respondent 3). These estimated PSQ scores and effects differ when other influencing background variables are present (Table 3, Figure 4).

**Table 3:** Effects of all the factors and the effects of a combination of factors with the intervention in men and women on the Pain Sensitivity Questionnaire scores.

Factor	Effect in women, β (95% CI)	*P* value	Effect in men, β (95% CI)	*P* value
(Intercept)	3.37 (2.98 to 3.76)	<.001	3.17 (2.75 to 3.59)	<.001
Film with contagious laughter vs neutral film	–.14 (–.56 to .28)	.52	–.19 (–.65 to .28)	.43
Film with painful scenes vs neutral film	.12 (–.29 to .053)	.57	.26 (–.20 to .72)	.27
Age (years)	.01 (0 to .01)	<.001	.01 (0 to .01)	<.001
BMI (kg/m^2^)	.01 (0 to .02)	.048	.01 (0 to .02)	.048
Smoking vs nonsmoking	–.23 (–.32 to –.13)	<.001	–.23 (–.32 to –.13)	<.001
Exercise 0-1 h/wk vs none	.13 (.02 to .25)	.02	.13 (.02 to .25)	.02
Exercise 2-3 h/wk vs none	0 (–.08 to .08)	.97	0 (–.08 to .08)	.97
Exercise 3-6 h/wk vs none	–.06 (–.16 to .05)	.30	–.06 (–.16 to .05)	.30
Exercise 7-10 h/wk vs none	–.27 (–.44 to –.09)	.003	–.27 (–.44 to –.09)	.003
Exercise >10 h/wk vs none	–.31 (–.63 to .01)	.06	–.31 (–.63 to .01)	.06
Secondary school vs primary school	–.10 (–.34 to .14)	.42	–.10 (–.34 to .14)	.42
Secondary vocational school vs primary school	–.14 (–.37 to .1)	.26	–.14 (–.37 to .1)	.26
Higher professional education or up vs primary school	–.36 (–.60 to –.13)	.002	–.36 (–.60 to –.13)	.002
No education vs primary school	.20 (–.18 to .57)	.29	.20 (–.18 to .57)	.29
NRS^a^ mild pain score 1-3 vs no pain	.07 (–.12 to .25)	.49	.26 (–.02 to .54)	.07
NRS moderate pain score 4-7 vs no pain	.72 (.55 to .89)	<.001	.65 (.33 to .96)	<.001
NRS severe pain score 8-10 vs no pain	1.32 (1.08 to 1.56)	<.001	1.01 (.46 to 1.55)	<.001
Duration of pain (shorter than 3 months vs no pain)	.01 (–.10 to .11)	.91	.01 (–.10 to .11)	.91
Duration of pain (longer than 3 months vs no pain)	–.05 (–.22 to .13)	.61	–.05 (–.22 to .13)	.61
Film with contagious laughter and NRS mild pain score 1-3	–.16 (–.41 to .09)	.22	–.05 (–.43 to .34)	.80
Film with painful scenes and NRS mild pain score 1-3	–.03 (–.28 to .23)	.82	–.23 (–.61 to .15)	.23
Film with contagious laughter and NRS moderate pain score 4-7	–.21 (–.45 to .02)	.08	.04 (–.38 to .45)	.87
Film with painful scenes and NRS moderate pain score 4-7	–.19 (–.44 to .05)	.12	–.10 (–.52 to .32)	.64
Film with contagious laughter and NRS severe pain score 8-10	–.28 (–.62 to .07)	.12	–.37 (–1.14 to .39)	.34
Film with painful scenes and NRS severe pain score 8-10	–.42 (–.76 to –.07)	.02	–.06 (–.80 to .67)	.86
Film with contagious laughter and BMI	.01 (–.01 to .02)	.26	.01 (–.01 to .02)	.26
Film with painful scenes and BMI	–.01 (–.03 to 0)	.07	–.01 (–.03 to 0)	.07

^a^NRS: numeric rating scale.

## Discussion

### Principal Results

Our study results indicate that determining whether men or women have a higher sensitivity to pain requires considering the influence of other variables. These factors have differential effects on the pain sensitivity in men and women. Differences in effects are even present in a simple, although not validated, intervention, showing the importance of always considering individual factors in treatment options and future studies. Only investigating the effect of an intervention and the influence of sex provides a limited perspective on the complex multidimensional problem of pain between sexes.

### Limitations

The cross-sectional analysis of demographics encompassed the entire study population, while randomization occurred before PSQ completion, resulting in a smaller group available for analyzing sex differences and the influence of background variables on the PSQ scores. Additionally, a lower number of respondents completed the questionnaire in this group compared to those who watched a short film first. In retrospect, the PSQ could have been filled in at baseline at the start of the web-based study and after viewing the film. Although we only included complete questionnaires, we lacked control over the interpretation of the respondents and completion of the questionnaires. Furthermore, an uneven distribution of sex, with more women participating, and a high percentage of respondents experiencing chronic pain may introduce bias. People with chronic pain are more likely to be interested and to participate in this project. We addressed this concern by conducting multivariable regression analyses to correct for potential confounders.

Within-sex differences in pain sensitivity linked to gender expression have been reported previously [25]. For example, men who consider themselves as more masculine show higher thresholds for pain [26]. It is therefore very plausible that gender expression has an effect on pain perception and treatment as well, but our data were not sufficient to support or reject this hypothesis. In the previous baseline questionnaires [25,26], respondents had the option to fill in their gender if their gender identity did not correspond to their biological sex. The number of respondents who identified with this option was low and the demographic data were inconclusive. Psychological and other socioeconomic factors were not considered in our study as well. It is plausible that these factors influence pain and interventions on pain, and we would recommend taking these factors into account in future research.

We used the nature film as a neutral (control) group because there were fewer respondents in the control group. The short films were composed via a discussion-based focus group through evoked emotions, but these films were not validated. At the time, we did not have access to validated sets of films. We wanted to make our study more appealing for respondents. When looking at the smaller number of completed questionnaires in respondents who had not seen a film, the supposed appeal of the film was correct. For further research, the group could be asked about the evoked emotions in our used films for validation.

### Statistical Analysis

In the case of our study, we had a set of independent variables that were potentially related with pain sensitivity. In case of redundant variables such as year of birth and age, full inclusion would give multicollinearity that can be eliminated without any downside. Either year of birth or age could be used. However, such redundancies are not present in the data. Overall, little multicollinearity is seen, with one exception. Pain duration and NRS score are correlated and show high variance inflation factors (above 10). This can be eliminated by removing either one of them from the model. However, in that case, no insights on the relation of the removed variable and pain sensitivity would be gained. Insights on the remaining variables would be confounded by the effects of the variable removed. Therefore, we decided to pay the price for allowing the multicollinearity.

### Comparison With Prior Work

Women have been regarded as the more pain-sensitive sex, with previous studies reporting heightened pain responses in females. Although the greater pain sensitivity in women is evident in specific experimental settings [27,28], our large cohort study does not report a higher pain sensitivity in women with use of the PSQ. Different background factors (BMI, highest level of education, smoking level, and current pain level) influence pain sensitivity differently in women compared with that in men, as investigated in our control group (n=1746). Experiencing pain is one of the most important risk factors for developing chronic pain in another site of the body, suggesting that the presence of pain influences pain sensitivity [8]. This is supported by our data. In women who experience moderate to severe pain, the PSQ scores were notably higher. Similarly, severe pain exerted a negative influence on pain sensitivity in men, although the limited sample size of this subgroup prevented definitive conclusions. These findings align with previous research, highlighting sensitization in individuals with chronic pain and elevated PSQ scores in such patients [21,29].

Smoking emerged as another influential factor, displaying distinct effects in women. This contradicts previous results, which show that smokers experience a higher pain intensity [30]. However, the overall impact of smoking on chronic pain states remains inconclusive, with modest evidence suggesting an association [31,32]. Although people with lower education levels often experience chronic pain more frequently and with greater intensity, our data did not show an effect of education level on pain sensitivity in women [8]. In men, higher education influences the PSQ score positively, leading to a lower score on the PSQ. This may support the notion that sociocultural characteristics affect men more than women [8,33]. Contrary to previous findings, our study did not observe a positive effect of physical activity [34].

In addition to investigating background factors, we examined the impact of watching a film on pain sensitivity. We found that respondents experiencing pain had a lower score on the PSQ after watching a film with painful situations (Table 2). An explanation could be that context can relieve pain and that the situations stated in the PSQ are considered more relative for the respondents to what is shown on the film [35]. This result is in contrast to the empathy for pain theory, in which respondents experience pain themselves when watching others endure a painful situation [36,37]. There was no observed effect of the other films, contrary to previously performed research in smaller numbers on the effect of social laughter or funny films on pain [18,38]. This may suggest that context plays a crucial role in pain relief. Participants were not in a social setting with no researcher present when viewing the film—a setting that is known to affect pain perception [39,40].

The effects of audiovisual stimuli were different between sexes when considering the effects of other background factors. Sex, BMI, and (the level of) current pain influence the effects of both the contagious laughter film and the painful event film. Sex, BMI, and current pain are both independent influential factors, but being a female with current pain had an extra lowering effect on the PSQ. A systematic review on obesity and pain sensitivity suggested a correlation between higher BMI and increased pain sensitivity, although the impact of an intervention and the role of BMI remain understudied [41].

### Conclusions

In our full cohort, the scores on the PSQ were not different between men and women. When exploring other factors, we do see a difference of their effects on the PSQ scores in men and women. These results underscore the importance of considering sex and sex-dependent background variables to obtain a comprehensive understanding of pain sensitivity. Hypotheses regarding pain sensitivity cannot be definitively accepted or rejected without accounting for these influential factors. Studying the effects of background factors in different treatment strategies could lead to further development of better individualized multimodal treatment for patients with pain, as has been proposed by other researchers [33,42-44].

## Abbreviations

CONSORT: Consolidated Standards of Reporting Trials
NRS: numeric rating scale
PSQ: Pain Sensitivity Questionnaire

## Data Availability

The data sets generated and analyzed during this study are available from the corresponding author on reasonable request.
